# Molecular characterization of the Great Lakes viral hemorrhagic septicemia virus (VHSV) isolate from USA

**DOI:** 10.1186/1743-422X-6-171

**Published:** 2009-10-25

**Authors:** Arun Ammayappan, Vikram N Vakharia

**Affiliations:** 1 Center of Marine Biotechnology, University of Maryland Biotechnology Institute, Baltimore, 701 East Pratt Street, Baltimore, Maryland 21202-3101, USA; 2 Department of Veterinary Medicine, University of Maryland, College Park, MD 20742, USA

## Abstract

**Background:**

Viral hemorrhagic septicemia virus (VHSV) is a highly contagious viral disease of fresh and saltwater fish worldwide. VHSV caused several large scale fish kills in the Great Lakes area and has been found in 28 different host species. The emergence of VHS in the Great Lakes began with the isolation of VHSV from a diseased muskellunge (*Esox masquinongy*) caught from Lake St. Clair in 2003. VHSV is a member of the genus *Novirhabdovirus*, within the family *Rhabdoviridae*. It has a linear single-stranded, negative-sense RNA genome of approximately 11 kbp, with six genes. VHSV replicates in the cytoplasm and produces six monocistronic mRNAs. The gene order of VHSV is 3'-N-P-M-G-NV-L-5'. This study describes molecular characterization of the Great Lakes VHSV strain (MI03GL), and its phylogenetic relationships with selected European and North American isolates.

**Results:**

The complete genomic sequences of VHSV-MI03GL strain was determined from cloned cDNA of six overlapping fragments, obtained by RT-PCR amplification of genomic RNA. The complete genome sequence of MI03GL comprises 11,184 nucleotides (GenBank GQ385941) with the gene order of 3'-N-P-M-G-NV-L-5'. These genes are separated by conserved gene junctions, with di-nucleotide gene spacers. The first 4 nucleotides at the termini of the VHSV genome are complementary and identical to other novirhadoviruses genomic termini. Sequence homology and phylogenetic analysis show that the Great Lakes virus is closely related to the Japanese strains JF00Ehi1 (96%) and KRRV9822 (95%). Among other novirhabdoviruses, VHSV shares highest sequence homology (62%) with snakehead rhabdovirus.

**Conclusion:**

Phylogenetic tree obtained by comparing 48 glycoprotein gene sequences of different VHSV strains demonstrate that the Great Lakes VHSV is closely related to the North American and Japanese genotype IVa, but forms a distinct genotype IVb, which is clearly different from the three European genotypes. Molecular characterization of the Great Lakes isolate will be helpful in studying the pathogenesis of VHSV using a reverse genetics approach and developing efficient control strategies.

## Background

Viral hemorrhagic septicemia virus (VHSV) is a rhabdoviral fish pathogen, which constitute one of the major threats to the development of the aquaculture industry worldwide. VHSV causes disease not only in salmonids, but also in many other marine species as well [1-5]. The virus usually causes severe hemorrhages on the skin, the kidney and the liver, with mortality rates as high as 90%. VHSV is a member of the genus *Novirhabdovirus *within the family *Rhabdoviridae *[6]. It possess a non-segmented negative-strand RNA genome of approximately 11 kbp with a coding capacity for five structural proteins; nucleoprotein (N), phosphoprotein (P), matrix protein (M), glycoprotein (G), RNA polymerase (L), and a nonstructural protein (NV) [7-9]. The gene order of VHSV is 3'-leader-N-P-M-G-NV-L-trailer-5'. The negative-strand RNA genome is connected tightly with the nucleoprotein N and forms the core structure of virion. This encapsidated genomic RNA is also associated with the phosphoprotein P and polymerase protein L, which are involved in viral protein synthesis and replication.

The complete nucleotide sequence of VHSV has been determined initially for VHSV Fi13 strain [9] and coding regions of several other strains of VHSV have been determined later [10]. In this study, we characterized the entire genome of the Great Lakes VHSV isolate MI03GL from muskellunge, *Esox masquinongy *(Mitchill), caught from the NW region of Lake St. Clair, Michigan, USA in 2003 [11]. Affected fish exhibited congestion of internal organs; the inner wall of the swim bladder was thickened and contained numerous budding, fluid-filled vesicles. Lake St. Clair is a major lake in the Great Lakes system that has historically supported an economically and socially important sport fishery for many species of fish [11,12]. VHSV has a very broad host-range, including numerous taxonomic families of fish. The Great Lakes VHSV has been found in 28 different host species, including muskellunge, yellow perch, smallmouth bass, northern pike, whitefish, walleye, bluegill, drum, round gobies, and some sucker species . It is a serious threat to all aquaculture species, including salmonids such as trout and salmon. To understand the molecular characteristics of the Great Lakes VHSV strain MI03GL, we thoroughly analyzed the entire genomic sequences and compared it with other VHSV strains and rhabdoviruses.

## Methods

### RT-PCR amplification of the VHSV genome

The genomic RNA of VHSV strain MI03GL was kindly provided by Dr. Gael Kurath, U.S. Geological Survey, Western Fisheries Research Center, Seattle, WA, and was used as a template. The consensus PCR primers were designed based on the available VHSV genome sequences (Genbank accession numbers AB179621; NC_000855; AB490792) from the National Center for Biotechnology Information (NCBI). The complete genome sequences were aligned; highly conserved sequence segments identified, and used to design overlapping primers. The oligonucleotide primers used in this study are listed in Table 1. First strand synthesis was carried out in a tube containing 5 μl of RNA, which was denatured at 70°C for 10 min in the presence of DMSO (3 μl), 1 μl forward gene-specific primer, 1 μl of 25 mM dNTPs, and snap-cooled on ice for 1 min. The reaction mixture containing 2 μl of 10× RT buffer, 2 μl of 0.1 M DTT, 4 μl of 25 mM MgCl_2_, 1 μl of Superscript III RT™, and 1 μl of RNase OUT™ was incubated at 50°C for 1 h. PCR amplifications were carried out using a *pfx*50™ PCR kit (Invitrogen, CA), according to manufacturer's instructions. Briefly, the following mixture was used for PCR amplification: 3 μ1 of cDNA, 2 μl of primer mix; 5 μl of 10× PCR buffer [100 mM Tris-HCl (pH 9.0), 500 mM KC1, 1% Triton X-100], 2 μ1 of 25 mM MgCl_2_, 0.5 ul of *pfx*50 polymerase, and 37 μ1 of DEPC water, to make a final volume of 50 μ1. Reaction was carried out in a thermal cycler (MJ Research Inc., Waltham, MA), using the following program: denaturation at 94°C for 30 sec; annealing for 30 sec at 60°C; and extension at 68°C for 2 min. The RT-PCR products were separated by agarose gel electrophoresis and purified using a QIAquick gel extraction kit (Qiagen, CA).

**Table 1:** Oligonucleotides used for cloning and sequencing of the VHSV genome

**VHSV primers**	**Sequences**	**Position**
VHSV 1F	GTATCATAAAATATGATGAGT	1-21

VHSV 1R	CAACTTGAACTTCTTCATGGC	2028-2008

VHSV 2F	AAGAAGACCGACAACATACTCT	1858-1879

VHSV 2R	GACGAAACTTTGAGAGGAGAAA	3993-3972

VHSV 3F	ATCTCATTACCAACATGGCTCAAA	3892-3915

VHSV 3R	TTGTTCGCTTCTCCCCTAATTGT	5932-5910

VHSV 4F	TGCCATAGACCTACTCAAGTTAT	5814-5835

VHSV 4R	CTGATCCATGGTGGCTATGTGAT	8042-8020

VHSV 5F	AGATGATTGTCTCCACCATGAA	7846-7867

VHSV 5R	GAGATCCGCTCTCGTTCATCAA	10027-10006

VHSV 6F	GACAAGAAAGCTGGGAAGAGA	9787-9807

VHSV 6R	GTATAGAAAATAATACATACCA	11183-11162

VHSV 850R	ACAGTCCAATCATGGTCATTC	851-831

VHSV 1MF	GGACAAAATGATCAAGTACATC	595-616

VHSV 2MF	CCATTCTCTGTGAAGATCAACAT	2456-2478

VHSV 3MF	TGTGAGACAGAAAGATGACGAT	4566-4587

VHSV 4MF	GACACCACCGAGAAGAGACTAC	6429-6450

VHSV 5MF	GAAGAGAAGGAAGCACACCAA	8424-8444

VHSV 5'End1	GTGGCATCCGTCTTTCTCAA	10599-10618

VHSV 5'End2	CGCTCATCACTCTCCTCGAA	10660-10679

Oligo (dT)	GCGGCCGCTTTTTTTTTTTTTTTTTTTTT	

In order to identify the 3'-terminal region of the genomic RNA, poly (A) tail was added to the 3'-end with poly (A) polymerase enzyme, according to manufactures' instruction (Applied Biosystems, USA). Tailing reaction was carried in a tube containing 30 μl of RNA, 26 μl of nuclease-free water, 20 μl of 5× poly (A) polymerase buffer, 10 μl of 25 mM MnCl_2_, 10 μl of 10 mM ATP, and 4 μl of *E. coli *poly (A) polymerase. The reaction mixture was incubated at 37°C for 1 hr and then RNA was purified using a Qiagen RNAeasy kit, according to manufacturer's instructions. The cDNA synthesis and polymerase chain reaction were conducted as described above, using an oligo (dT) primer (5'-GCGGCCGCTTTTTTTTTTTTTTTTTTTTT-3') for the first-strand synthesis, followed by PCR with the VHSV-specific primer 850R (5'-ACAGTCCAATCATGGTCATTC-3'). The 5'-terminal of genomic RNA was identified by rapid amplification of the 5'-end, using a 5'RACE kit (Invitrogen, USA), according to manufacturer's instructions.

### Cloning and sequencing

The purified RT-PCR products were cloned into a pCR2.1 TOPO^® ^TA vector (Invitrogen, CA). Plasmid DNA from various clones was sequenced by dideoxy chain termination method, using an automated DNA sequencer (Applied Biosystems, CA). Three independent clones were sequenced for each amplicon to exclude errors that can occur from RT and PCR reactions.

### Sequence and phylogenetic tree analysis

The assembly of contiguous sequences and multiple sequence alignments were performed with the GeneDoc software [13]. The pair-wise nucleotide identity and comparative sequence analyses were conducted using Vector NTI Advance 10 software (Invitrogen, CA) and BLAST search from NCBI. Phylogenetic analyses were conducted using the MEGA4 software [14]. Construction of a phylogenetic tree was performed using the ClustalW multiple alignment algorithm and Neighbor-Joining method with 1000 bootstrap replicates.

### Database accession numbers

The complete genome sequence of the VHSV MI03GL strain was submitted to the GenBank (accession number GQ385941). The accession numbers of other viral sequences used for sequence comparison and phylogenetic analysis are listed in Table 2.

**Table 2:** Information about the viral hemorrhagic septicemia virus (VHSV) isolates used in this study for comparison and phylogenetic analysis

**S. No**	**Strain**	**Country**	**Host**	**GenBank no**.
**N protein**				

1.	07-71	France	VHSV-infected cell line EPC	D00687

2.	Makah	USA	Coho salmon	X59241

**P protein**				

3.	07-71	France	rainbow trout	U02624

4.	Makah	USA	Coho salmon	U02630

**M protein**				

5.	Makah	USA	Coho salmon	U03503

6.	07-71	France	rainbow trout	U03502

**G protein**				

7.	NO-2007-50-385	Denmark	rainbow trout	EU547740

8.	Dwb97-04	Germany	rainbow trout	EU708816

9.	Datt107	Germany	rainbow trout	EU708734

10.	Au917-04	Austria	rainbow trout	EU708733

11.	Au28-95	Austria	rainbow trout	EU708729

12.	JF00Ehi1	Japan	Japanese flounder	AB490792

13.	BC99-001	Canada	Pacific sardine	DQ401195

14.	BC99-010	Canada	Pacific herring	DQ401194

15.	ME03	Canada	Atlantic herring	DQ401192

16.	JP99Obama25	Japan	Japanese flounder	DQ401191

17.	JP96KRRV9601	Japan	Japanese flounder	DQ401190

18.	WA91Clearwater	USA	coho salmon	DQ401189

19.	BC99-292	Canada	Atlantic salmon	DQ401188

20.	BC93-372	Canada	Pacific herring	DQ401186

21.	BC98-250	Canada	Atlantic salmon	DQ401187

22.	KRRV9822	Japan	Japanese flounder	AB179621

23.	UK-MLA98/6PT11	North Sea	Norway pout	AY546632

24.	UK-MLA98/6HE1	North Sea	herring	AY546631

25.	UK-H17/5/93	North Sea, E. Shetland	cod	AY546630

26.	UK-H17/2/95	North Sea, E. Shetland	haddock	AY546629

27.	UK-860/94	Gigha, W Scotland	turbot	AY546628

28.	SE-SVA32	Kattegat	Bottom-living*	AY546627

29.	SE-SVA31	Kattegat	herring	AY546626

30.	NO-A16368G	Norway	rainbow trout	AY546621

31.	IR-F13.02.97	Ireland	turbot	AY546620

32.	GE-1.2	Georgia	rainbow trout	AY546619

33.	FR-L59X	France	Eel	AY546618

34.	FR-2375	France	rainbow trout	AY546617

35.	FI-ka422	Gulf of Bothnia	rainbow trout	AY546615

36.	DK-200079-1	Denmark	rainbow trout	AY546613

37.	DK-200098	Denmark	rainbow trout	AY546605

38.	DK-9895174	Denmark	rainbow trout	AY546603

39.	DK-2835	Denmark	rainbow trout	AY546585

40.	DK-5123	Denmark	rainbow trout	AY546588

41.	DK-5e59	Denmark	dab	AY546583

42.	DK-1p8	Denmark	herring	AY546573

43.	CH-FI262BFH	Switzerland	rainbow trout	AY546571

44.	AU-8/95	Austria	rainbow trout	AY546570

45.	DK-1p52	Denmark	sprat	AY546576

46.	AY167587	Korea	olive flounder	AY167587

47.	Cod Ulcus	UK	Atlantic cod	Z93414

48.	Hededam	Denmark	rainbow trout	Z93412

49.	96-43	UK	Atlantic herring	AF143862

50.	Fil3	France	rainbow trout	Y18263

51.	02-84 France	France	Salmo trutta	VHU28800

52.	Makah	USA	Coho salmon	VHU28747

53.	FA281107	Norway	rainbow trout	EU481506

**NV protein**				

54.	DK-1p55	Baltic Sea	Sprat	DQ162801

55.	DK-1p53	Baltic Sea	herring	DQ159195

56.	DK-1p52	Baltic Sea	Sprat	DQ159194

57.	DK-1p49	Baltic Sea	rockling	DQ159193

58.	F1	Denmark	rainbow trout	U47848

59.	07-71	France	rainbow trout	U28746

60.	Makah	USA	Coho salmon	U28745

**Complete genome**				

61.	JF00Ehi1	Japan	Japanese flounder	AB490792

62.	FA281107	Norway	rainbow trout	EU481506

63.	Fil3	France	rainbow trout	NC_000855

64.	KRRV9822	Japan	Japanese flounder	AB179621

65.	Cod Ulcus	UK	Atlantic cod	Z93414

66.	Hededam	Denmark	rainbow trout	Z93412

67.	96-43	UK	Atlantic herring	AF143862

68.	14-58	France	rainbow trout	AF143863

69.	07-71	France	rainbow trout	AJ233396

**Rhabdoviruses Complete Genome**				

70.	**Rhabdovirus**			**GenBank no.**

71.	Bovine ephemeral fever virus (BEFV)			NC_002526

72.	European bat lyssavirus (Bat)			NC_009527

73.	Northern cereal mosaic virus (Cereal)			NC_002251

74.	Lettuce necrotic yellows virus (Lettuce)			NC_007642

75.	Maize Fine streak virus			NC_005974

76.	Maize mosaic virus (MMV)			NC_005975

77.	Mokola virus			NC_006429

78.	Orchid fleck virus (OFV)			NC_009609

79.	Rabies virus			NC_001542

80.	Siniperca chuatsi rhabdovirus			NC_008514

81.	Spring viremia of carp virus (SVC)			NC_002803

82.	Sonchus yellow net virus (SYN)			NC_001615

83.	Taro vein chlorosis virus (Taro)			NC_006942 NC_006942 NC_006942

84.	Tupaia rhabdovirus			NC_007020

85.	Vesicular stomatitis virus (VSV)			NC_001560

86.	Infectious hematopoietic necrosis virus (IHNV)			X89213

87.	Hirame rhabdovirus (HIRRV)			NC_005093

88.	Snakehead rhabdovirus (SHRV)			NC_000903

*Virus was isolated from pool of *Pholis gunellus*, *Gobiidae *species, *Zoarces viviparous *and *Acanthocottus scorpius*.

## Results

### Complete nucleotide sequence of the VHSV strain MI03GL

**Figure 1:**
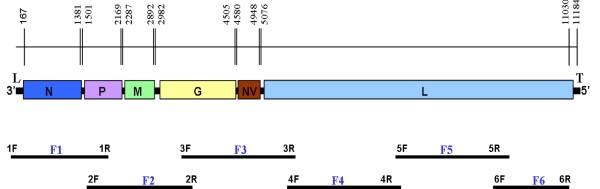
**Genetic map of the VHSV genome and cDNA clones used for sequence analysis**. The location and relative size of the VHSV ORFs are shown; the numbers indicate the starts and ends of the respective ORFs. Six cDNA fragments (F1 to F6) were synthesized from genomic RNA by RT-PCR. The primers used for RT-PCR fragments are shown at the end of each fragment. The RNA genome is 11,184 nucleotides long and contains a leader (L) and trailer (T) sequences at its 3'-end and 5'-end, respectively. The coding regions of N, P, M, G, NV and L genes are separated by intergenic sequences, which have gene-start and gene-end signals.

The entire genome of VHSV-MI03GL strain was amplified as six overlapping cDNA fragments that were cloned, and the DNA sequenced (Fig. 1). The complete genome sequence of VHSV-MI03GL comprises 11,184 nucleotides (nts) and contains six genes that encode the nucleocapsid (N) protein, the phosphoprotein (P), the matrix protein (M), the glycoprotein (G), the non-virion (NV) protein, and the large (L) protein (Fig. 1). The gene order is similar to other novirhabdoviruses, 3'-N-P-M-G-NV-L-5'. The genomic features and predicted proteins of the VHSV strain MI03GL are shown in Table 3. All the open reading frames (ORFs) are separated by untranslated sequences, known as gene junctions, whereas the untranslated regions at the 3'- and 5'- ends are known as the 'leader' and 'trailer', respectively. For example, the N gene is composed of 1,388 nts, and is located between 54 and 1441 nts from the 3'-end of the genomic RNA. The ORF of N gene is flanked by 113 nts and 60 nts of 5'- and 3'-untranslated regions (UTRs), respectively, and encodes a protein of 404 amino acids, with a calculated molecular weight (MW) of 44.0 kDa. Similarly the length, ORF, and UTRs of the P, M, G, NV, and L genes, encoding respective proteins with their calculated MW, are depicted in Table 3.

**Table 3:** Genomic features and predicted proteins of the VHSV strain MI03GL

**S. No**	**Gene**	**Start**	**End**	**5'UTR**	**ORF**	**3'UTR**	**Total Length**^**a**^	**Protein Size (aa)**	**MW**^**b**^
1.	Leader	1	53				53		

2.	N	54	1441	113	1215	60	1388	404	44.0

3.	P	1444	2203	57	669	34	760	222	24.4

4.	M	2206	2946	81	606	54	741	201	22.3

5.	G	2949	4556	33	1524	51	1608	507	56.9

6.	NV	4559	4979	21	369	31	421	122	13.6

7.	L	4982	11068	94	5955	38	6087	1984	224.1

8.	Trailer	11069	11184				116		

^a ^Total length of a gene including 5'UTR, ORF and 3'UTR

^b ^Predicted molecular weight of proteins in kilodaltons (kDa)

### Genomic termini and untranslated sequences

**Figure 2:**
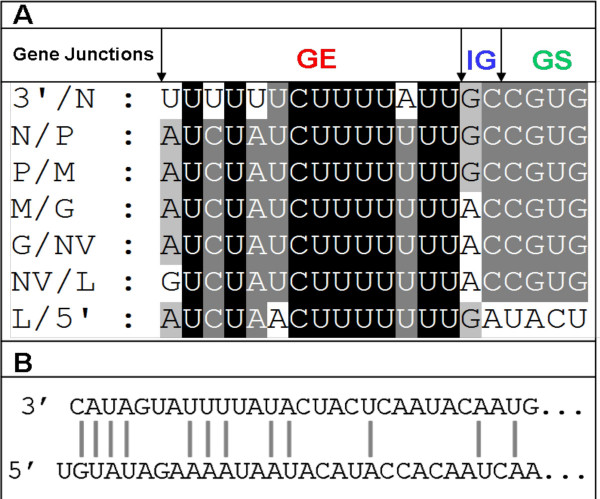
**Analysis of the gene junctions and complementarities in the VHSV genome**. **A**) Seven identified gene junctions of VHSV in the negative-sense of the genomic RNA are shown. 3'/N, junction of 3'-leader and nucleocapsid gene; N/P, junction of nucleocapsid and phosphoprotein gene; P/M, junction of phosphoprotein and matrix gene; M/G, junction of matrix and glycoprotein gene; G/NV, junction of glycoprotein and non-virion gene; NV/L, junction of non-virion and polymerase gene; L/5'-, junction of polymerase gene and 5' trailer. GE = Gene end; IG = Intergenic di-nucleotide; GS = Gene start. **B**)Complementarities of the 3'- and 5'-ends of the VHSV genome. The first 4 nucleotides of 3'-end are complementary to the 5'-end nucleotides of genomic RNA, except an additional uracil (U) residue at the 5'-terminal.

Rhabdoviruses have conserved untranslated regions between open reading frames for optimal translation of viral proteins [15]. These sequences consist of a putative transcription stop/polyadenylation motif (UCUAUCU^7^), which signals reiterative copying of the U sequences to generate poly (A) tail to the mRNA. It is followed by an intergenic di-nucleotide GC or AC, which is not transcribed, and a putative transcription start signal, -CGUG- (Fig. 2A). All the genes contain these conserved gene end (GE), intergenic (IG) and gene start (GS) sequences, as shown in Fig. 2A.

Like other rhabdoviruses, the genomic termini of VHSV 3'-terminal nucleotides exhibit complementarities to the nucleotides of the genomic 5'-terminus. Figure 2B shows that the first 4 nucleotides of 3'-end are complementary to the 5'-end nucleotides of genomic RNA, with the exception of an additional uracil (U) residue at the 5'-terminal. The complementary nature of genomic termini allows a formation of a panhandle structure, which is important for replication of rhabdoviruses.

### Homology and phylogenetic analysis

The percent nucleotide and deduced amino acid sequence identities of VHSV-MI03GL with known VHSV strains and other rhabdoviruses were determined by Vector NTI program and the results are shown in Tables 4 and 5, respectively. The complete genome comparison of MI03GL with other VHSV strains reveals a close relationship with two Japanese strains, which were isolated from Japanese flounder [JF00Ehi1 (96%) and KRRV9822 (95%)]. Other VHSV strains are only 86-87% identical to the MI03GL strain (Table 4). Similarly, the complete genome comparison of MI03GL strain with different members of *Rhabdoviridae *family shows 30-35% identity, but among novirhabdoviruses, it exhibits 56% identity with infectious hematopoietic necrosis virus (IHNV) and 62% with snakehead rhabdovirus (SHRV), as shown in Table 5. Also in novirhabdoviruses, it is evident that non-virion protein (which is absent in other rhabdoviruses) is highly variable than any other region of the genome, showing only 16-17% identity.

**Table 4:** Percent (%) nucleotide or deduced amino acid sequence identity of the Great Lakes VHSV-MI03GL with other VHSV strains ^a, b, c^

**VHSV Strains**	**3'UTR**^**¥**^	**N**	**P**	**M**	**G**	**NV**	**L**	**5'UTR**^**¥**^	**Complete** **Genome**^**¥**^
07-71	95	92	90	**97**	93	73	78	79	86

Fi13	95	92	93	**96**	93	74	**96**	80	87

FA281107*	95	92	94	**96**	94	72	**96**	76	87

**JF00Ehi1**	**96**	**96**	**100**	**98**	**96**	**89**	**99**	**90**	**96**

**KRRV9822**	**94**	**97**	**94**	**98**	**95**	**90**	**96**	**87**	**95**

14-58	-	93	93	**96**	94	74	**96**	-	87^┼^

96-43	-	93	94	**98**	93	75	**97**	-	87^┼^

Cod Ulcus	-	93	94	**97**	94	74	**97**	-	87^┼^

Hededam	-	93	94	**97**	94	76	**97**	-	87^┼^

**Makah**	-	**94**	**98**	**98**	**96**	**92**	-	-	-

DK-1p49	-	-	-	-	-	72	-	-	-

DK-1p53	-	-	-	-	-	72	-	-	-

DK-1p55	-	-	-	-	-	72	-	-	-

DQ159194	-	-	-	-	-	72	-	-	-

^a ^bold letters in rows and columns indicates VHSV strains and VHSV proteins showing highest identity with MI03GL strain

^b¥ ^only nucleotide sequences were used for analysis

^c ^*termini sequences were incomplete; ^┼ ^only coding sequences were available for comparison; (-) denotes that sequences are not available

**Table 5:** Percent (%) nucleotide or deduced amino acid sequence identity of the VHSV strain MI03GL with other rhabdoviruses

**Rhabdoviruses**	**3'UTR**^**¥**^	**N**	**P**	**M**	**G**	**NV**	**L**	**5'UTR**^**¥**^	**Complete** **genome**^**¥**^
BEFV	39	8	12	8	13	NA	13	36	32

Cereal	27	11	9	8	10	NA	13	28	30

Bat	38	9	11	10	18	NA	15	32	35

Maize Fine streak	31	8	8	10	7	NA	13	32	30

Lettuce	27	11	11	8	8	NA	12	38	30

MMV	30	10	14	10	8	NA	13	25	32

Mokola	41	10	8	12	19	NA	14	38	34

OFV	27	8	7	2	7	NA	13	32	NA

Rabies	38	10	11	9	16	NA	15	34	35

Siniperca	34	8	7	8	13	NA	15	30	31

SVC	35	9	8	5	17	NA	14	35	34

SYNV	29	8	12	9	6	NA	13	22	30

Taro	26	10	12	9	10	NA	14	33	32

Tupaia	30	9	8	10	14	NA	15	44	31

VSV	38	9	8	5	13	NA	15	32	34

**IHNV**	**35**	**40**	**35**	**36**	**38**	**16**	**60**	**35**	**56**

**HIRRV**	**32**	**39**	**34**	**38**	**38**	**17**	**59**	**34**	**56**

**SHRV**	**52**	**46**	**42**	**45**	**48**	**16**	**65**	**37**	**62**

^¥ ^Only nucleotide sequences were used for analysis

NA, not applicable

BEFV, Bovine ephemeral fever virus; Bat, European bat lyssavirus; MMV, Maize mosaic virus; Cereal, Northern cereal mosaic virus; Lettuce, Lettuce necrotic yellows virus; OFV, Orchid fleck virus; SYNV, Sonchus yellow net virus; SVC, Spring viremia of carp virus; Taro vein chlorosis virus (Taro); VSV, Vesicular stomatitis virus; IHNV, Infectious hematopoietic necrosis virus; HIRRV, Hirame rhabdovirus; SHRV, Snakehead rhabdovirus.

-Viruses belonging to *Novirhabdovirus *genus are in bold letters

**Figure 3:**
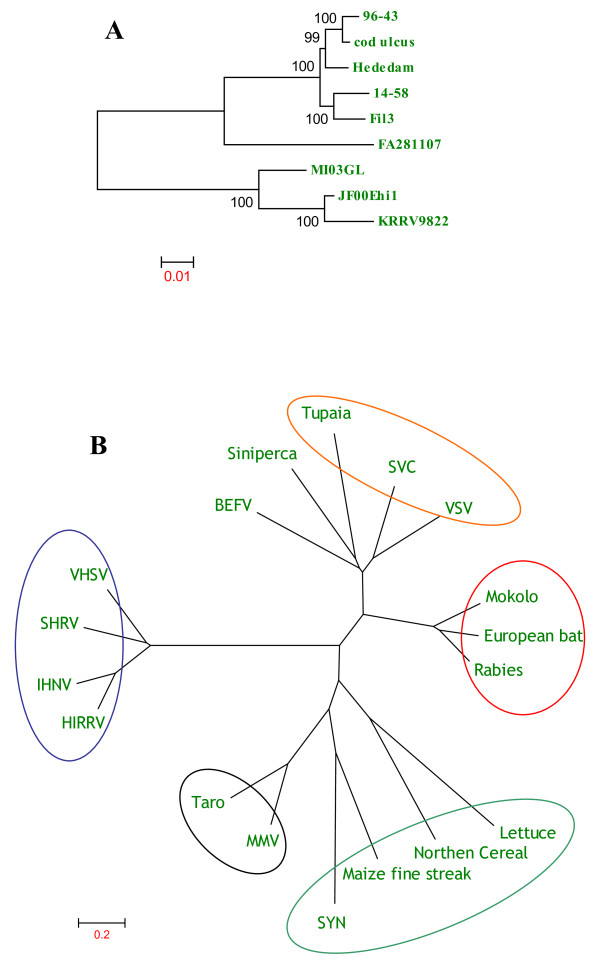
**Phylogenetic tree analysis of the deduced amino acid sequences of VHSV (A) and various other rhabdovirus genomes (B)**. Information about the VHSV strains and rhabdoviruses sequences used in this analysis is described in Table 2. Rhabdoviruses belonging to the same genus are circled in B. *Novirhabdovirus *(Blue); *Lyssavirus *(Red); Vesiculovirus (Orange); *Cytorhabdovirus *(Teal); *Nucleorhabdovirus *(Black); BEFV-*Ephemerovirus*; Siniperca-unclassified rhabdovirus. Phylogenetic tree analysis was conducted by neighbor-joining method using 1000 bootstrap replications. The scale at the bottom indicates the number of substitution events and bootstrap confidence values are shown at branch nodes.

Figure 3 shows the phylogenetic trees generated by comparing the deduced amino acid sequences of VHSV strains and other rhabdoviruses belonging to *Rhabdoviridae *family. Phylogenetic tree obtained by comparing the deduced amino acid sequences of VHSVs shows that MI03GL strain is closely related to the Japanese strains, JF00Ehil and KRRV9822 (Fig. 3A), whereas phylogenetic tree obtained by comparing the deduced amino acid sequences of known rhabdoviruses reveals that viruses belonging to the same genera of *Vesiculovirus, Lyssavirus, Ephemerovirus, Novirhabdovirus, Cytorhabdovirus*, and *Nucleorhabdovirus *would form separate clusters (Fig. 3B).

**Figure 4:**
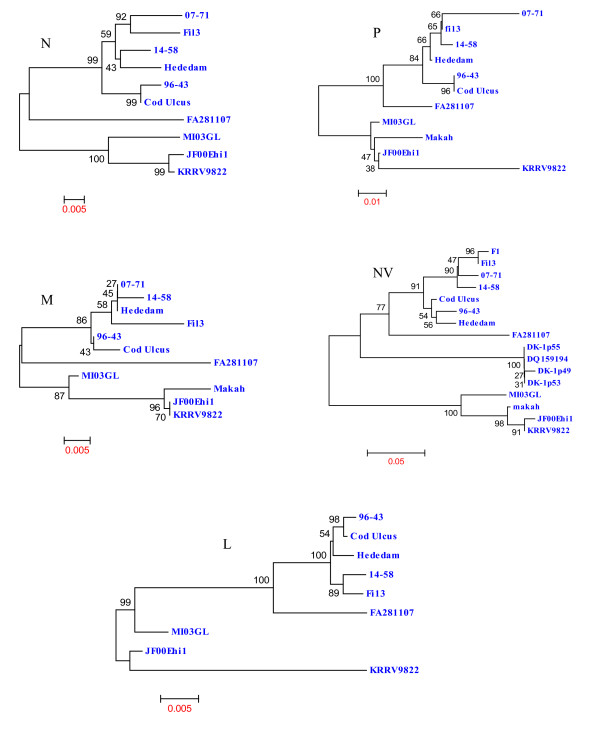
**Phylogenetic tree analysis of the deduced amino acid sequences of nucleocapsid (N), matrix (M), phosphoprotein (P), non-virion protein (NV) and polymerase protein (L) of various VHSV strains**. Information about the VHSV strains used in this analysis is described in Table 2. Phylogenetic tree analysis was conducted by neighbor-joining method using 1000 bootstrap replications. The scale at the bottom indicates the number of substitution events and bootstrap confidence values are shown at branch nodes.

**Figure 5:**
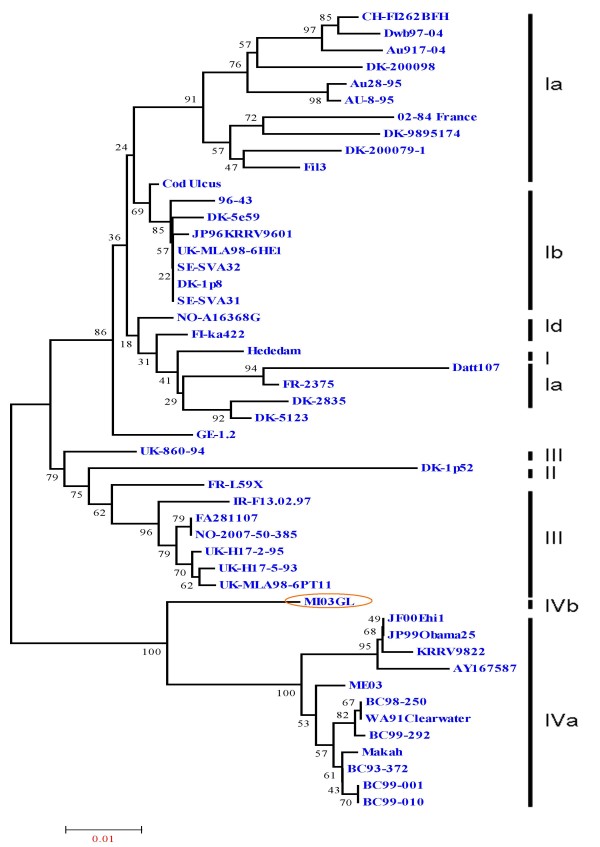
**Phylogenetic relationship of the full-length glycoprotein (G) sequences of 48 VHSV strains**. Genotypes and sublineages are depicted by bold vertical lines, as described by Einer-Jensen et al. (2004) and Elsyad et al., 2006. The Great Lakes strain MI03GL (circled) forms different sublineage IVb, whereas rest of the North American VHSV isolates falls under sublineage IVa. Data of virus isolates used here are shown in Table 2. Phylogenetic tree analysis was conducted by neighbor-joining method using 1000 bootstrap replications. The scale at the bottom indicates the number of substitution events and bootstrap confidence values are shown at branch nodes.

Figure 4 shows the phylogenetic trees formed by comparing the deduced amino acid sequences of MI03GL strain N, P, M, NV and L proteins with other VHSV strains, in which it is apparent that MI03GL proteins clusters with JF00Ehi1, KRRV9822 and Makah VHSV strains, except the L protein. Figure 5 shows the phylogenetic tree obtained by comparing 48 glycoprotein gene sequences of different VHSV strains, in which MI03GL clusters with subtype IVa members but forms a distinct clade, IVb.

## Discussion

The Great Lakes strain of VHSV (MI03GL) was isolated from muskellunge, *Esox masquinongy *(Mitchill), in 2003 from Lake St. Clair, Michigan, USA. Previously, only G and N protein gene sequences for MI03GL strain were available and sequence analysis of the G gene revealed that it is closely related to the North American genotype IVa but distinct from the three European genotypes [11]. To fully understand the molecular characteristics of the Great Lakes VHSV, we determined the complete genome sequence of MI03GL strain. The genome is 11,184 nts long and the gene organization (N, P, M, G, NV and L) is similar to all members of the *Novirhabdovirus *genus. The termini of the viral genome have conserved sequences at the 3'-end (CAUAG/UU) and 5'-end (G/AAUAUG) as other members of the *Novirhabdovirus *genus. The first 4 nt of the leader sequence VHSV are complementary to the last 4 nt sequence of the trailer region (Fig 2B). The length of the 3' leader of MI03GL is 53 nts, which is similar to SHRV but slightly shorter than IHNV and hirame rhabdovirus (HIRRV; 60 nts). VHSV has the longest 5' trailer (116 nts) than other novirhabdoviruses, such as SHRV (42 nts), IHNV (102 nts), and HIRRV (73 nts). It is possible that the difference in length of trailer sequences may have some functional significance, which remains to be seen.

All the genes of VHSV start with a conserved gene start sequence (-CGUG-) like other novirhabdoviruses, followed by an ORF and conserved gene-end sequence (A/GUCUAU/ACU^7^). All the genes end with 7 uracil (U) residues, which are poly adenylation signal for polymerase when it transcribes a gene. Polymerase adds poly (A) by stuttering mechanism [16]. After this poly (A) signal, there are two conserved intergenic di-nucleotides (G/AC), which are untranscribed and act as spacers between the two genes. Polymerase skips these two nucleotides to next gene-start sequence and starts transcribing the next gene [16]. Transcription of rhabdovirus mRNAs is regulated by cis-acting signals located within the 3' leader region and untranslated region between each gene ORF [17-20]. The Kozak context for each gene is conserved and all the genes have adenosine (A) nucleotide at -3 position before the start codon (data not shown). Among all the genes, L gene has the optimal Kozak context (-ACCATGG-) as only few copies of the L mRNA are produced inside the cell, and every single mRNA has to be utilized efficiently to make polymerase protein that is essential for both replication and transcription.

Comparison of the available VHSV sequences indicates the presence of 5 highly variable regions (HVRs) in the N protein: I, 38-54; II, 76-87; III, 98-131; IV, 367-375 and V, 391-393. Phylogenetic tree of the N protein shows clustering of MI03GL, JF00Ehil, KRRV9822 and Makah strains. The major variation between MI03GL and rest of above said three strains is in HVR I and IV (data not shown). The N-terminal half of the P protein of VHSV is highly variable, whereas C-terminal half is conserved. Phylogenetic tree of the P protein shows clustering of MI03GL, JF00Ehil and Makah strains. The strain isolated from Japanese flounder, JF00Ehil is 100% identical to the MI03GL. The highly conserved nature of phosphoprotein demonstrates its importance in viral replication. The matrix (M) protein is an important structural component of virions, forming a layer between the glycoprotein containing outer membrane and the nucleocapsid core. Matrix protein of VHSV is highly conserved than any other protein. VHSV strains used in this study exhibit very close (96-98%) identity with MI03GL. In phylogenetic analysis, JF00Ehil, KRRV9822 and Makah strains form a cluster, which is 99-100% identical to each other, and 98% identical to MI03GL. Matrix protein of rhabdovirus is involved in viral assembly, condensation of nucleocapsid, formation of bullet-shaped virion [21,22] and induces apoptosis by shutdown of host cell machinery in infected cells [23,24]. Because it is highly essential for assembly and release of virions, the matrix protein maintains highest homology between VHSV strains than any other protein.

The non-virion protein (NV) of VHSV shows greatest genetic diversity than any other proteins of VHSV (Table 4). It was demonstrated that NV-knockout IHNV replicates very slowly in cell culture and is non-pathogenic in fish [25]. On the contrary, NV-knockout SHRV replicates very well as wild-type virus and it was shown that NV protein of SHRV is not essential for pathogenesis [26]. These studies suggested that each species of *Novirhabdovirus *genus has its own characteristics and one can not ignore the importance NV in pathogenesis. The wide host-range for VHSV suggests that the tropism and the pathogenicity not only reside in glycoprotein gene, but also in other genes, especially the NV gene. The L protein displays the highest level of sequence homology among members of various genera of *Rhabdoviridae *family (Table 5). All the available L sequences for VHSV strains show highest conservation (98%) as that of the matrix protein.

Genomic comparison of VHSV strains isolated from various marine species from different parts of the world sheds light on the correlation of genetic sequences with viral tropism and pathogenicity. The glycoprotein is believed to be involved in virulence and tropism because of it's involvement in viral attachment and cell entry [27]. Comparison of the glycoproteins of various VHSV strains has revealed only few blocks of conserved region (data not shown). The regions between residues 53-70; 140-156; 232-253 and 389-413, are highly conserved and the rest of the region shows genetic variations which are scattered all over the protein. The major neutralizing epitopes have been mapped to two antigenic sites for IHNV, at amino acids 230-231 and 272-276 [28,29]. In this analysis, we found no amino acid substitutions at positions 230-231 among 48 strains compared, except two. On the other hand, residues 270-281 are highly variable, which supports earlier findings and suggests the involvement of this site in antigenic variation and virulence [30].

In phylogenetic analysis of the G proteins, MI03GL forms a separate branch in genotype IVa (Fig. 5) and is sub-typed as IVb, as demonstrated earlier [11]. Although JF00Ehil, KRRV9822 and Makah strains maintain close identity with MI03GL, they are sub-typed as IVa. The genogroups of VHSV are determined based on the restriction fragment length polymorphism patterns of the G protein [31]. Makah maintains a close identity with Japanese JF00Ehil (99%) and KRRV9822 (98%), and North American isolates (99%). Phylogenetic tree of the G protein explicitly demonstrates the relationship of Makah strain with members of genotype IV. Makah strain isolated from Coho Salmon in 1988 from Washington, USA was grouped under genotype IVa [31]. Rests of the North American strains belonging to genotype IVa were isolated in different time periods (1991-2003) [11], and Japanese strains were isolated around year 2000. Isolates of genotype IV have been recovered mainly in North America, Japan and Korea [31,32] but not in Europe where genotypes I, II and III are prevalent. It was suggested that VHSV strains circulating in a defined geographical area have a remarkably conserved G gene, regardless of the elapsed time or the different host species [33]. These earlier reports and the current study suggests that the genotype IV strains of VHSV probably originated from North America and possible ancestor for isolates of genotype IV might be Makah. This suggests that MI03GL might have diverged from Makah and evolved independently thereafter. To date, among VHSV strains, MI03GL strain is the only member of the genotype IVb.

## Competing interests

The authors declare that they have no competing interests.

## Authors' contributions

VNV conceived the study. AA planned the experimental design and carried out cloning and sequencing. AA drafted the manuscript. All authors critically reviewed and approved the final manuscript.
